# *In Vivo* Identification and Characterization of CD4^+^ Cytotoxic T Cells Induced by Virulent *Brucella abortus* Infection

**DOI:** 10.1371/journal.pone.0082508

**Published:** 2013-12-19

**Authors:** Anna Martirosyan, Kristine Von Bargen, Vilma Arce Gorvel, Weidong Zhao, Sean Hanniffy, Johnny Bonnardel, Stéphane Méresse, Jean-Pierre Gorvel

**Affiliations:** 1 Aix-Marseille University, CIML, 13288, Marseille, France; 2 CNRS, UMR 7280, 13288, Marseille, France; 3 INSERM, U631, 13288, Marseille, France; National University, Costa Rica

## Abstract

CD4^+^ T cells display a variety of helper functions necessary for an efficient adaptive immune response against bacterial invaders. This work reports the *in vivo* identification and characterization of murine cytotoxic CD4^+^ T cells (CD4^+^ CTL) during *Brucella abortus* infection. These CD4^+^ CTLs express granzyme B and exhibit immunophenotypic features consistent with fully differentiated T cells. They express CD25, CD44, CD62L ,CD43 molecules at their surface and produce IFN-γ. Moreover, these cells express neither the co-stimulatory molecule CD27 nor the memory T cell marker CD127. We show here that CD4^+^ CTLs are capable of cytolytic action against *Brucella*-infected antigen presenting cells (APC) but not against *Mycobacterium*-infected APC. Cytotoxic CD4^+^ T cell population appears at early stages of the infection concomitantly with high levels of IFN-γ and granzyme B expression. CD4^+^ CTLs represent a so far uncharacterized immune cell sub-type triggered by early immune responses upon *Brucella abortus* infection.

## Introduction

CD4^+^ T cells are usually described as « helper » cells. They have a major role in controlling and regulating the immune system by providing help for B-cell activation and generation of cytotoxic CD8^+^ T cells [13,14]. According to functional properties and cytokine secretion profiles, CD4^+^ T cells are grouped into different subsets that provide a homeostatic balance between pro and anti-inflammatory responses. Helper T cells heterogeneity was first discovered two decades ago with the description of T_H_1 and T_H_2 cells [13,14]. T_H_1 cells are involved in host defense against intracellular pathogens and tumor cells by producing high levels of IFN-γ, TNF-α and IL-2. T_H_2 cells are responsible for coordinating humoral immunity, eosinophilic inflammation and are involved in host defense against extracellular parasites by secreting IL-4, IL-5, IL-10 and IL-13. New distinct subsets (T_H_9, T_H_22, T_H_17, T_H_FH, NKT, Treg cells) have been described confirming the heterogeneity of the CD4^+^ T cell family [15,16]. Several studies in mice and humans have reported a cytotoxic potential of CD4^+^ T cells. These initial observations have usually been restricted to cell lines and CD4^+^ T cell clones generated by *in vitro* culture [17-20]. The cytotoxic activity of CD4^+^ T cells has therefore often been considered as an artefact [21]. However, recent reports described CD4^+^ cytotoxic T cells (CTL) in peripheral blood, i.e. *ex vivo*, in various human pathologies such as Human Immunodeficiency Virus (HIV) [22], Cytomegalovirus (CMV) [23], poxvirus [24], Influenza [25] and Epstein - Barr virus (EBV) infections [26], B-cell chronic lymphocytic leukemia [27] and rheumatoid arthritis [28]. Healthy individuals have few CD4^+^ CTLs, representing 2% of the whole CD4^+^ T cell population. This percentage is markedly increased in HIV infection, with up to 50% of CD4^+^ T cells exhibiting a cytotoxic potential [22,29]. Moreover, cytolysis of virally infected cells by CD4^+^ T cells has been observed *in vivo* in lymphochoriomeningitis virus (LCMV) [30] and gamma-herpes virus [31] infected mice. CD4^+^ CTLs could represent a novel CD4^+^ subset with a unique lineage and functionality. The immune activation may thus be a major factor driving CD4^+^ T cell differentiation and promoting the acquisition of lytic properties [32-35]. 

*Brucella* is an intracellular bacterial pathogen that causes abortion and infertility in mammals and leads to a debilitating febrile illness that can progress into a long severe complications in humans [1]. *Brucella* species are closely related and display very similar pathogenic behavior, although they vary in their virulence and host affinity. For humans, the most pathogenic species are *Brucella melitensis*, *Brucella abortus* and *Brucella suis* [2]. Immune response to *Brucella* infection depends on the host, species or strain of *Brucella* and environment [3]. Moreover due to practical, economical and ethical reasons, studying brucellosis in natural hosts is difficult. In this context, mouse models have been widely used and have provided important mechanistic insights into the understanding of immune response against virulent *Brucella* [4,5].

Although innate immunity is capable of controlling *Brucella* replication during the acute phase in mice, effective adaptive immunity is necessary to mount a strong immune response at later stages of infection [6,7]. It has been largely described that IFN-γ-mediated T_H_1 responses are essential for the clearance of the pathogen [6]. Indeed, IFN-γ produced by CD4^+^ T cells and γδ T cells have recently been shown to activate the bactericidal properties in macrophages to hamper *Brucella* intra-host survival whereas IFN-γ produced by CD8^+^ T cells and B cell-related humoral immunity are modest players against *Brucella* infection in mice [8,9] although it has been demonstrated that T_H_1-type antibody isotypes such as IgG2a and IgG3, opsonize *Brucella* to facilitate phagocytosis and bacterial delivery into degradative endocytic compartments [10]. In humans, IFN-γ producing CD4^+^ T cells, CD8^+^ T cells and γδ T cells have been implicated in the control of brucellosis [11,12].

In *Brucella* infection it has been proposed that CD4^+^ T cells display a cytotoxic potential upon *Brucella* vaccination [36] and it has been shown that perforin -/- mice have a decreased ability to control *B. abortus* replication at early stages of infection [7]. 

Here we have studied cell-mediated adaptive immune responses during *Brucella abortus* infection. We have identified CD4^+^ CTLs in the mouse model of virulent *B. abortus* infection. CD4^+^ CTLs produce high levels of cytotoxic factor Granzyme B, express immunophenotypic features consistent with fully differentiated T cells and display cytolytic activity against infected phagocytes. We propose that CD4+ CTLs represent an immune cell-subtype triggered by early immune responses upon *Brucella abortus* infection.

## Results

### Identification of GranzymeB^+^CD4^+^ T cells upon *Brucella* infection

**Figure 1:**
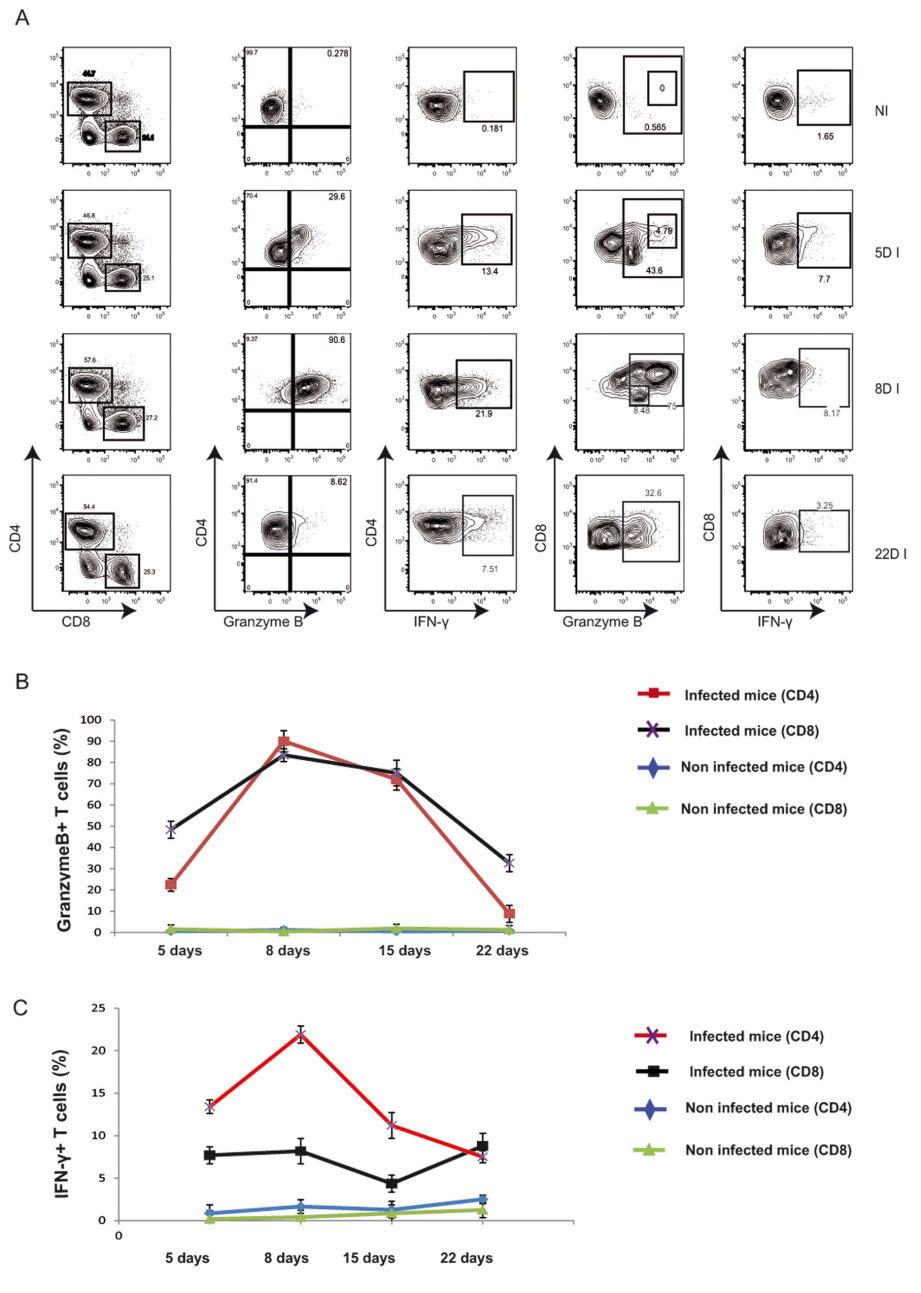
Identification of a GranzymeB^+^ CD4^+^T cell population upon *Brucella* infection. (A) CD4^+^ and CD8^+^ T cells from *Brucella*-infected C57BL/6 mouse spleens were analyzed by flow cytometry for the synthesis of IFN-γ and Granzyme B at 5, 8 and 22 days post-infection. Numbers in outlined areas indicate percentage of cells. Data are representative of 3 separate experiments each involving groups of 5 mice. Results of one representative experiment showing the percentages of CD4^+^ and CD8^+^ T cells synthesizing Granzyme B (B) and IFN-γ (C) in infected and non-infected mice.

**Figure 2:**
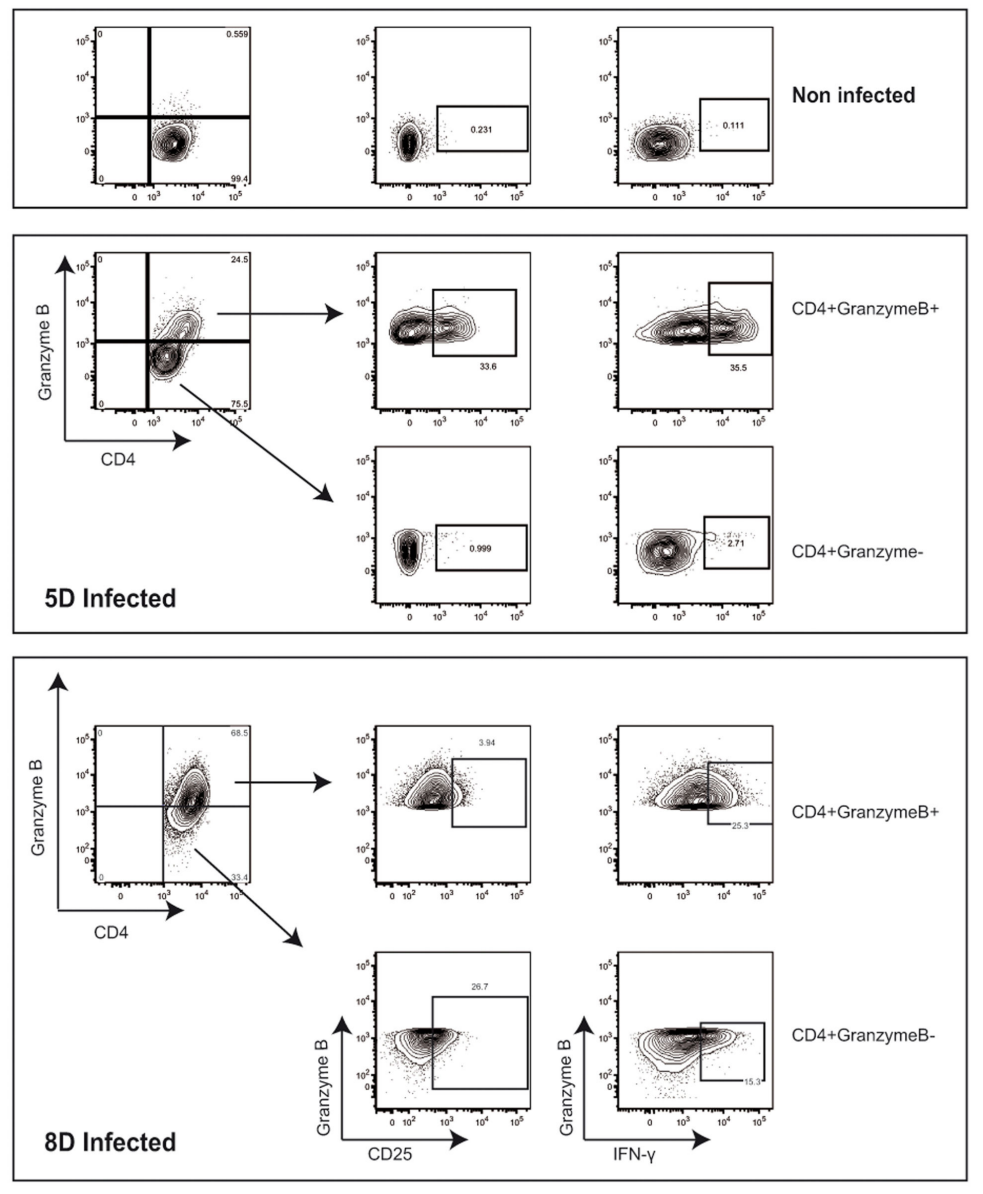
Immunophenotypic characterization of CD4^+^ CTLs upon *Brucella* infection. Granzyme B-expressing CD4^+^ T cells from *Brucella*-infected C57BL/6 mice were analyzed by flow cytometry for the expression of CD25 and the production of IFN-γ at 5 and 8 days post-infection. Numbers in outlined areas indicate percentages of cells. Data are representative of 3 separate experiments each involving groups of 5 mice.

To study the cellular immune responses generated during *Brucella* infection we examined CD4^+^ and CD8^+^ T cell activation at several time points post-infection by measuring the surface expression of CD25, CD44 and the intracellular synthesis of effector molecules such as Granzyme B and IFN-γ. At 5 days post-infection, the CD8^+^ T cell population in the spleen expressed high levels of Granzyme B (Figure 1) and was able to synthesize IFN-γ (Figure 1A and C). Interestingly, Granzyme B expression was also observed in 20% of CD4^+^ T cells (Figure 1). Cellular immune responses were even more pronounced at 8 days post-infection. Approximately 80% of the CD8^+^ T cell population expressed Granzyme B and strikingly almost the entire CD4^+^ T cell population was positive for Granzyme B (Figure 1A and B), 20% of which synthesized IFN-γ (Figure 1A and C). The percentage of Granzyme B-positive CD4^+^ varied between 70-90% depending on experiments (Figures 1 and 2). This is the first *in vivo* demonstration of such a high population of CD4^+^ T cells with a cytotoxic potential. CD4^+^ and CD8^+^ T cell responses persisted in the spleen up to 15 days post-infection, (Figure 1B). We also observed that T cell activation decreased at 22 days post-infection together with a strong decline of the Granzyme B-expressing CD4^+^ T cell population (Figure 1B).

T cell responses were also investigated in various lymph nodes (LNs) such as mesenteric LNs (MLNs), axillary LNs (ALNs) and inguinal LNs (ILNs). The pattern of cellular immune responses was similar in these different LNs. CD4^+^ and CD8^+^ T cell responses were triggered in the different LNs at 8 days post-infection, as illustrated in ALNs (Figure S1). Both, CD4^+^ and CD8^+^ T cells up-regulated the expression of CD25 (not shown) and produced effector molecules such as Granzyme B and/or IFN-γ (Figure S1). In ALNs, 27% of the whole CD4^+^ T cell population expressed Granzyme B (Figure S1). This population was lower in ILNs and MLNs, representing 5% and 13%, respectively (not shown). From 15 days post-infection onwards, T cell responses in the LNs decreased (Figure S1) and we could not detect any CD4^+^ Granzyme B^+^ T cell subset, showing that the induction of Granzyme B-expressing CD4^+^ T cells correlates with the triggering of CD4^+^ and CD8^+^ T cell responses. 

BALB/c mice have been shown to be more sensitive to *Brucella* infection than C57BL/6 mice [1]. A large sub-population of CD4^+^ T cells expressing Granzyme B was also identified in BALB/c mice infected with *Brucella* (Figure S2A). 

Overall, these data suggest that *Brucella* infection triggers a subset of CD4^+^ Granzyme B^+^ T cells with a cytotoxic potential. 

### Characterization of Granzyme B^+^CD4^+^ T cells upon *Brucella* infection

We then performed a detailed phenotypic analysis using a panel of markers in order to characterize the Granzyme B^+^CD4^+^ T cell subsets. At 5 days post-infection, Granzyme B^+^CD4^+^ T cells up-regulated the adhesion molecule CD44, but expressed neither the co-stimulatory receptor CD27 nor the memory T cell marker CD127 (a subunit of interleukin-7 receptor-α) (Figure S3A). Moreover, Granzyme B^+^CD4^+^ T cells expressed CD25 (the cell surface α chain of IL-2 receptor) and synthesized IFN-γ (Figure 2). At 8 days post-infection, the expression of CD44 and CD25 by GranzymeB^+^CD4^+^ T cells was strongly decreased, whereas IFN-γ production persisted (Figures 2 and S3B). These data show that the population of Granzyme B^+^CD4^+^ T cells changed their surface expression repertoire during different stages of infection. A strong inflammatory response was characterized by a high up-regulation of CD43 and a down-regulation of CD62L in both CD4^+^ and CD8^+^ T cells at 8 days post-infection (not shown). Moreover, Granzyme B^+^CD4^+^ T cells were neither regulatory T cells (Foxp3^-^) nor NKT cells (negative for the natural killer (NK) cell-associated marker NK1.1, (CD161) (Figure S2B). These immunophenotypic features are consistent with fully differentiated T cells. 

### Bacterial load and IFN-γ secretion during infection

**Figure 3:**
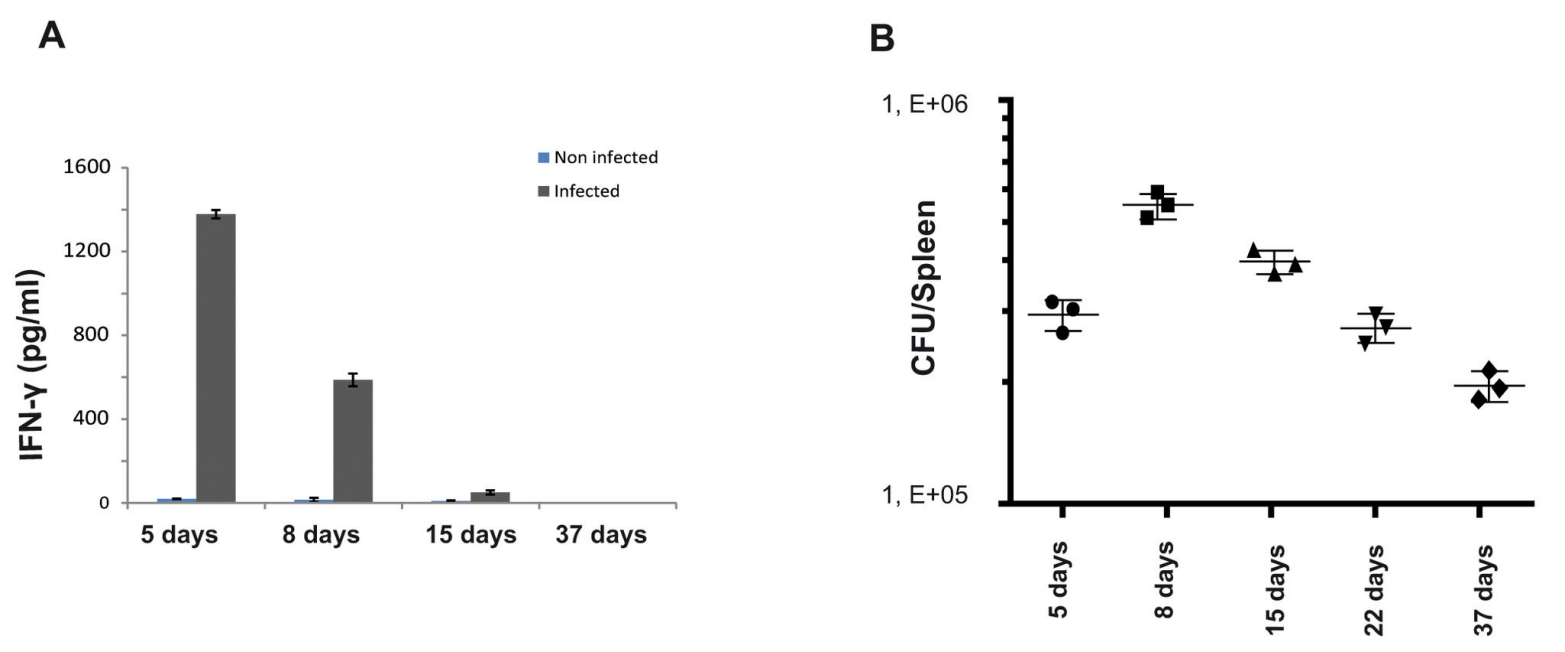
Bacterial loads and IFN-γ production in *Brucella* infected mice. Mice were infected intraperitoneally with 10^6^ *B. abortus* 2308. At 5, 8, 15, 22 and 37 days post-infection, the level of IFN-γ was determined in the sera of mice intraperitoneally infected with *Brucella abortus* (A). CFUs were determined in the spleens of C57BL/6 infected mice (B). Data are representative of three independent experiments each involving groups of 3 mice.

It has been largely described that IFN-γ-mediated T_H_1 responses are essential for the clearance of the pathogen [6]. We have therefore measured the amount of IFN-γ in the sera of infected mice. The highest levels were observed at 5 days post-infection and then decreased from 8 days post-infection onwards (Figure 3A). Moreover we have observed the secretion of very low levels of TNF-α (not shown). The cytokine expression was also analysed by QRT-PCR in the spleen of 8 and 15 days infected mice. The induction of IFN-γ, IL-6 and Granzyme B was detected (not shown).

We next analyzed the bacterial load in the spleen (Figure 3B) of mice infected with *B. abortus*. The significant decrease (p<0.01) of the *Brucella* bacterial load from 8 days post-infection onwards (Figure 3B) correlated with a very strong T cell response, a large Granzyme B-expressing CD4^+^ T cell population (Figure 1) and a diminution of IFN-γ secretion (Figures 3A). These data suggest that Granzyme B-expressing CD4^+^ T cells may participate in the adaptive immune responses induced during the initial phases of *B. abortus* infection. 

### Functional characterization of Granzyme B^+^CD4^+^T cells

**Figure 4:**
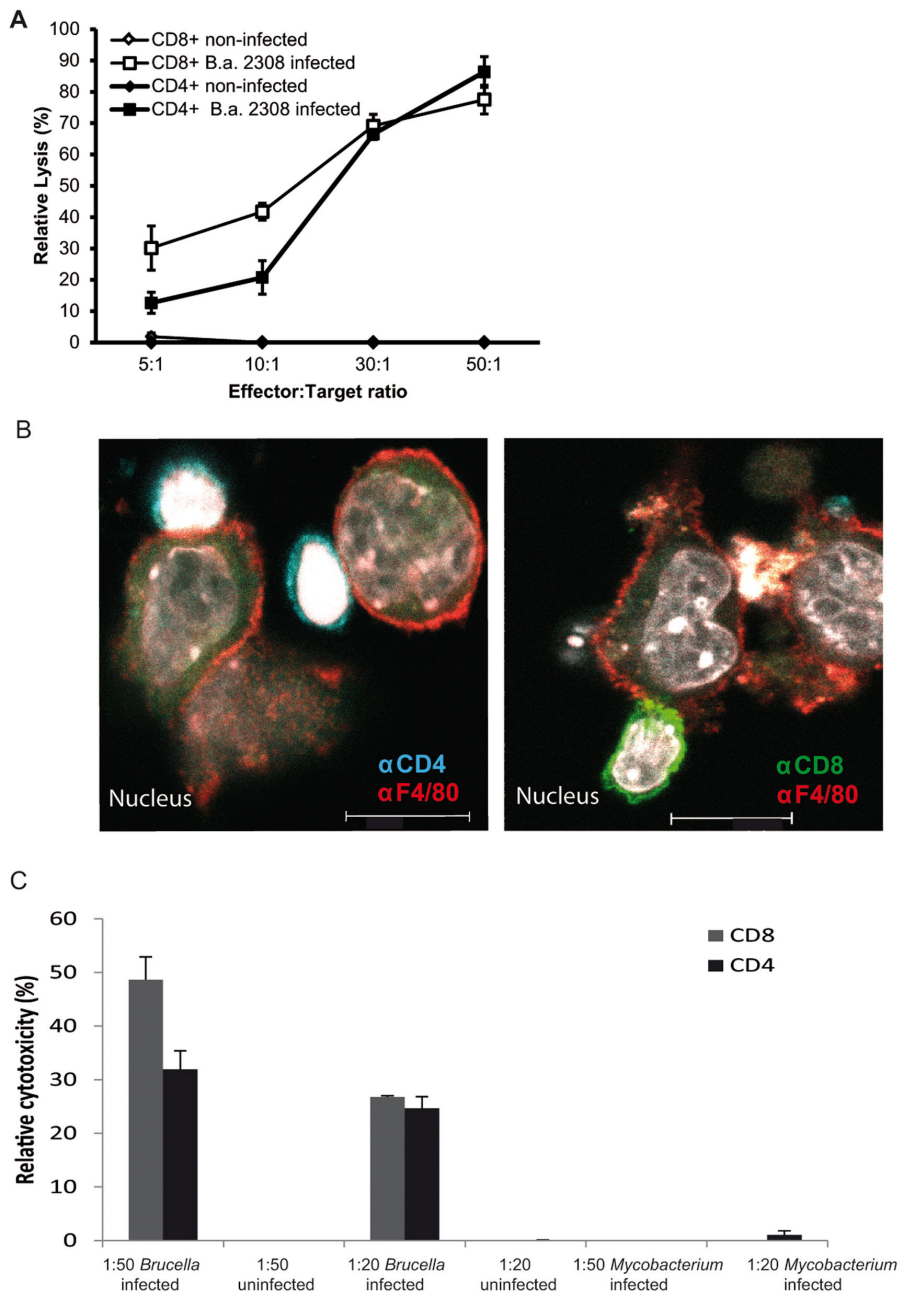
Cytolytic capacity of Granzyme B^+^CD4^+^T cells. Lysis of non-infected and *B. abortus*-infected macrophages in the presence of CD4^+^ and CD8^+^ T cells isolated from the spleens of BALB/c mice infected for 8 days with *Brucella* was analysed. Macrophage targets and effector cells were mixed at several effector:target /T ratios and incubated for 4 h. Values given represent relative macrophage lysis as compared to a Tx-100 treated 100% control (A). Parallel samples of heat-killed *B. abortus*-infected Raw macrophages on coverslips were processed for immunofluorescence microscopy. Representative micrographs show macrophages after 4 h of coculture with CD8^+^ or CD4^+^ effector cells (B). (C) Lysis of non-infected, *B. abortus*-infected and *M. avium*-infected macrophages in the presence of CD4^+^ and CD8^+^ T cells isolated from the spleens of BALB/c mice infected for 8 days with *Brucella* was analysed. Macrophage targets and effector cells were mixed at 1:50 and 1:20 target:effector ratios and incubated for 4 h. Values given represent relative macrophage lysis as compared to a Tx-100 treated 100% control.

To study the cytolytic capacities of Granzyme B^+^CD4^+^T cells generated during *Brucella* infection, we determined their cell-mediated cytotoxicity by an LDH-release assay. CD8^+^ and CD4^+^ T cells purified from *Brucella* infected mice were co-cultured at several ratios with macrophages uninfected or infected with *Brucella*. Cytotoxicity was measured by detecting the activity of LDH released from damaged cells. Results show that cell lysis by CD8^+^ and CD4^+^ T cells was specifically restricted to infected macrophages (Figure 4A). At low effector/target ratio, CD4^+^ T cells induced a less efficient killing of infected macrophages than CD8^+^ T cells. However, at higher ratios (30:1 and 50:1) CD4-mediated cytotoxicity was similar to that induced by CD8^+^ T cells (Figure 4A). Macrophages were also incubated with heat-killed *B. abortus* and co-cultured with either CD8^+^ or CD4^+^ effector cells and processed for either cytotoxicity assay (Figure S4) or immunofluorescence microscopy (Figure 4B). To further support the obtained results, parallel samples of macrophages incubated with heat-killed *B. abortus* and co-cultured with either CD8^+^ or CD4^+^ effector cells for 4 h were fixed and processed for immunofluorescence microscopy. Both CD8^+^ and CD4^+^ lymphocytes were observed in close contact with infected target cells some of which showed clear signs of nuclear fragmentation (Figure 4B). 

We next determined whether the lysis of target cells by Granzyme B^+^ CD4^+^ T cells is antigen-specific. In order to do so, effector cells from *Brucella*-infected mice were co-cultured either with macrophages infected with *Brucella* or with another intracellular bacterium, *Mycobacterium avium* (Figure 4C). Results show that effector cells from mice infected with *Brucella* efficiently lysed macrophages infected with *B. abortus*, whereas *M. avium*-infected macrophages were not affected. 

In addition, confocal microscopy shows (Figure S5) that Granzyme B in CD4^+^ T cells colocalized with Lamp-1, a marker for late endosome/lysosomes in *Brucella* infected macrophages. 

## Discussion

We identified for the first time a population of murine CD4^+^ CTLs during virulent *Brucella abortus* infection *in vivo*. This population produces high levels of Granzyme B and displays specific cytolytic capacity against infected macrophages. These cells correspond to fully differentiated CD4^+^ T cells and do not belong to the CD4^+^ regulatory T cell or to the CD4^+^ NKT cell subset. The immunophenotypic features of CD4^+^ CTLs include the expression of CD25, CD44, CD62L and CD43 molecules, as well as the production of IFN-γ. Moreover, cytotoxic CD4^+^ T cells expressed neither the co-stimulatory molecule CD27 nor the memory T cell marker CD127. The early phase of *Brucella* infection has been characterized as a T_H_1 (producing IFN-γ) type adaptive immune response [1] and CD4^+^ CTLs may participate in these first steps.

Here we show that at early stages of *Brucella* infection a heterogeneous effector T cell population including CD8^+^ CTLs and CD4^+^CTLs both expressing high levels of Granzyme B is present in the spleen and LNs, whereas CD4^+^Granzyme B^-^ IFN-γ^+^ T cells develop at later stages of the infection. Moreover, the significant decrease of the bacterial load in the spleen from 8 days post-infection onwards of *Brucella* infection coincides with the presence of a large CD4^+^ CTLs population, a strong T cell response and a diminution of pro-inflammatory cytokine secretion. Depletion experiments or adoptive transfer experiments of Granzyme B-expressing CD4^+^ T-cells into infected mice depleted of either CD4^+^ and CD8^+^ mice or using infected NOD scid IL2 receptor gamma chain knockout mice (NSG) may help to evaluate the importance of these cells in controlling pathogens.

So far, very little is known about the phenotype, function and transcriptional profile of cytolytic CD4^+^ T cells. For instance, it is not known when CD4^+^ T cells commit to the cytolytic CD4^+^ T-cell program during their lineage development. Furthermore, it is currently unknown if cytolytic activity is simply a function acquired by terminally differentiated CD4^+^ T_H_1 cells or Tregs, or if it represents a characteristic of a unique CD4^+^ T-cell subset. The multiparametric analysis of the various CD4^+^ T-cell phenotypes that have shown cytolytic potential, in combination with detailed microarray profiling will shed further light on this question. The identification of specific markers or transcriptional profiles may allow for a better assessment of cytolytic CD4^+^ T-cell responses and therefore help to evaluate their role in different disease settings. 

On the basis of our data and previous reports on chronic viral infection models, we propose that CD4^+^ CTLs represent another cell sub-type triggered by immune responses upon *Brucella* infection in addition to CD8^+^ effector and γδ T cells. The expansion of this subset occurs concurrently with the increase of CD8^+^ CTLs numbers. The same factors that trigger CD8^+^ T cell differentiation could drive the increase of CD4^+^Granzyme B^+^ T cells. Moreover, based on the similarities of cytolytic CD4^+^ T cells to T_H_1 and CD8^+^ T cells, the transcriptional factors Tbet, Eomes and Runx3 may play important roles in inducing this activity. It is also possible that γ-chain cytokines such as IL-15 and IL-21 may participate in the generation of cytolytic CD4^+^ T-cell responses [33]. 

The physiological role of CD4^+^ CTLs in humans is not yet understood, mainly due to the limitation of available tools. Their precise function in the development and maintenance of immunity against pathogens remains to be established. In this context, their identification in mouse models represents an important advance. It will provide a basis to study in detail the role of CD4^+^ T-cell differentiated subsets, in particular CD4^+^ CTLs and their importance in the immune control of bacterial infections. 

## Materials and Methods

### Ethics statement

Animal experimentation was conducted in strict accordance with good animal practice as defined by the French animal welfare bodies (Law 87–848 dated 19 October 1987 modified by Decree 2001-464 and Decree 2001-131 relative to European Convention, EEC Directive 86/609). All animal work was approved by the Direction Départmentale des Services Vétérinaires des Bouches du Rhône (authorization number 13.118). INSERM guidelines have been followed regarding animal experimentation (authorization No. 02875 for mouse experimentation).

### Mice and cells

Six weeks old female C57Bl/6 and BALB/c mice from Jackson Laboratory were used. INSERM guidelines have been followed regarding animal experimentation (authorization No. 02875 for mouse experimentation). Animals were killed by cervical dislocation at appropriate times. Cells were isolated from lymphoid organs (spleen and lymph nodes) of infected and non-infected mice using a mix of type I collagenase (Sigma) and DNAse I (Sigma-Aldrich). Isolated cells were stimulated by CD3 antibody for 4 h at 37°C in the presence of BD Golgi Stop (BD Biosciences). The expression of molecular markers, intracellular cytokines and Granzyme B was then evaluated by flow cytometry. Five mice were used for each infection and the representative data is shown. All experiments were carried out at least 3 independent times.

### Bacterial infection and replication assays

Virulent *B. abortus* strain 2308 was used for infection experiments. *Brucella* was grown in tryptic soy broth (TSB; Sigma-Aldrich). For infection, we inoculated 2 ml of media and bacteria were cultivated for 16 h at 37 °C up to an optical density (OD_600nm_) of approximately 2.0. Groups of 3 six-week-old female mice were intraperitoneally infected with 10^6^ CFU of *B. abortus* 2308. To calculate bacterial multiplication rates, spleens and lymph nodes were aseptically removed and individually homogenized in PBS. Dilutions from these homogenates were seeded on TSA and CFU numbers were determined. 

### Flow cytometry

Antibodies used for flow cytometry included PerCP-Cy5.5- CD4, Alexa700-CD8, Pacific blue-CD5, FITC-CD25, PE-CD127, PerCP-Cy5.5- CD27, Alexa700-CD44, PE-Cy7-CD4, FITC-NK1.1 (all from eBiosciences). The Aqua Dead Cell Stain (Invitrogen) was used to stain the dead cells. CD4 and CD8 T cells were fixed and permeabilized with Fixation/Permeabilization buffers (eBiosciences) following manufacturer’s protocol. PE-Cy7- IFN-γ (eBiosciences) and APC-Granzyme B (Invitrogen) antibodies were used for the intracellular labeling of lymphocytes. Isotype matched controls were used appropriately. At least 100,000 events were collected on flow cytometry Canto II (BDBiosciences). Flow cytometry data was analyzed by FlowJo software.

### Cytokine measurement

IFN-γ and TNF-α were quantified in the sera of *Brucella* infected mice (3 per group) by sandwich enzyme-linked immunosorbent assays (ELISA) according to the manufacturer’s instruction (eBiosciences). 

### Cytotoxicity assay

Balb/c mice were infected for 8 days with *B. abortus* 2308. CD4^+^ and CD8^+^ T cells from spleen were then negatively enriched using mouse CD8 and CD4 negative isolation kit (Dynal). CD4^+^ and CD8^+^ T cell percentages were determined by flow cytometry, the degree of purification obtained was > 90%. Raw macrophages were infected either with alive or heat killed *B. abortus* 2308 or alive *Mycobacterium avium* for 4 h and rinsed twice. Infected or uninfected macrophages were cultured alone (low control) or co-cultured with effector cells at a ratio effector: macrophage of 5:1, 10:1, 30:1 or 50:1 during 4 h. A 100% control of complete macrophage lysis (high control) was obtained by lysing corresponding wells with 1% TritonX-100. Cytotoxicity was determined by analyzing supernatants with the LDH-release assay (Roche Diagnostics) was then performed with the supernatants to determine cytotoxicity. Relative cytotoxicity was calculated as follows: Relative cytotoxicity (%) = [(Effector and target cell mix) – (Effector cells alone) – (low control)]/ (high control – low control)] x 100. The infection of target cells was confirmed by plating.

### Confocal fluorescence microscopy

Raw macrophages were seeded on coverslips, infected with *B. abortus* as described and incubated with CD4^+^ effector cells from the spleen of a mouse that had been infected with *B. abortus* for 8 days. After 1 h, samples were fixed in 3.2% formaldehyde in PBS. Residual formaldehyde was quenched by a 20 min incubation in 50 mM NH_4_CL in PBS. Fixed cells were permeabilized in fixation/permeabilization buffer from the Foxp3 / Transcription Factor Staining Buffer Set (eBioscience), rinsed once in permeabilization buffer (PB) of the same kit and blocked for 1h with PB containing 5% BSA. Coverslips were incubated in rabbit anti-LAMP1 at 1:1000 (obtained from obtained from Dr. Minoru Fukuda (La Jolla Cancer Research Foundation)) and anti-GranzymeB-APC at 1:100 (Invitrogen) in PB/5% BSA for 1h, rinsed twice in PB and subsequently stained in anti-rabbit-Cy3 (1:500), anti- CD4-FITC (1:100, Biolegend) and anti-mouse-Cy5 (1:500, Jackson ImmunoResearch) in PB/5% BSA for 1 h. Samples were rinsed twice in PB, once in PBS, once in H_2_O and embedded in ProLongGold (Invitrogen) for analysis with a confocal Leica SP5X microscope.

 For cytotoxicity assay, Raw macrophages were seeded onto coverslips and incubated with heat-killed *B. abortus* 2308 at an MOI of 100 for 4 h. Macrophages were rinsed twice with PBS. CD4^+^ or CD8^+^ effector cells isolated as described above were added at an effector:macrophage ratio of 5:1 and cells were incubated for another 4 h before fixation. Antibodies used for immunofluorescence staining were anti-CD8-Alexa488, anti-CD4-Alexa647, anti-F4/80 (all from Biolegend) and anti-rat-DyLight549 (Abcam). Cells’ nuclei were visualized by embedding samples into Prolong Gold (Invitrogen) containing DAPI. Coverslips were analysed at a Leica SP5X confocal microscope and images processed using the corresponding software LAS AF Lite. 

### Statistical analysis

All experiments were carried out at least 3 independent times. Statistical analysis was done using two-tailed unpaired Student's *t* test and all the results correspond to the means +/- standard errors. Differences were considered as significante when *P* values were <0.05. 

## Supporting Information

Figure S1 **Identification of a GranzymeB^+^ CD4^+^T cell population in the axillary lymph nodes (ALNs) of *Brucella*-infected mice.** CD4^+^ and CD8^+^ T cells from *Brucella*-infected C57BL/6 mice ALNs were analyzed by flow cytometry for the synthesis of IFN-γ and Granzyme B at 8 and 15 days post-infection. Numbers in outlined areas indicate percentages of cells. Data are representative of 3 separate experiments each involving groups of 5 mice.
(TIF)

Figure S2 **GranzymeB^+^ CD4^+^T cell population upon *Brucella* infection.** (A) CD4^+^ and CD8^+^ T cells from the spleens of BALB/c mice infected by *Brucella* were analyzed by flow cytometry for the synthesis of IFN-γ and Granzyme B at 5 and 8 days post-infection. (B) Granzyme B-expressing CD4^+^ T cell population from *Brucella*-infected C57BL/6 mice was analyzed by flow cytometry for the expression of Foxp3 and NK1.1 at 8 days post-infection. Numbers in outlined areas indicate percentages of cells. Data are representative of 3 separate experiments each involving groups of 5 mice.
(TIF)

Figure S3 **Characterization of CD4^+^ CTLs in *Brucella* infection model.** The Granzyme B-expressing CD4^+^ T cell population from *Brucella*-infected C57BL/6 mouse spleens was analyzed by flow cytometry for the expression of CD44, CD127 and CD27 at 5 and 8 days post-infection. Numbers in outlined areas indicate percentages of cells. Data are representative of 3 separate experiments each involving groups of 5 mice.
(TIF)

Figure S4 **Functional characteristic of GranzymeB^+^ CD4^+^T cells.** Macrophages were incubated with heat-killed *B. abortus* and cocultured with either CD8^+^ or CD4^+^ effector cells isolated from the spleens of BALB/c mice infected for 7 days with *Brucella*. Macrophage targets and effector cells were mixed at several effector:target /T ratios and incubated for 4 h. Effector cell-mediated cytotoxicity was analyzed by an LDH-release assay. Values given represent relative macrophage lysis as compared to a Tx-100 treated 100% control.
(TIF)

Figure S5 **CD4 T-cell Granzyme B mostly localizes in LAMP1-positive vesicles.** *B. abortus*-infected Raw macrophages were incubated for 1h with CD4 positive effector cells from the spleen of a mouse that had been infected with *B. abortus* for 8 days, fixed and processed for confocal fluorescence microscopy. Samples were stained for LAMP1, Granzyme B and CD4. White bar: 5 µm.
(TIF)
