# Supplementary figures and images for: In Vivo Identification and Characterization of CD4+ Cytotoxic T Cells Induced by Virulent Brucella abortus Infection
Source: PLoS One. 2013 Dec 19;8(12):e82508. doi: 10.1371/journal.pone.0082508 (PMC3868576; doi:10.1371/journal.pone.0082508)

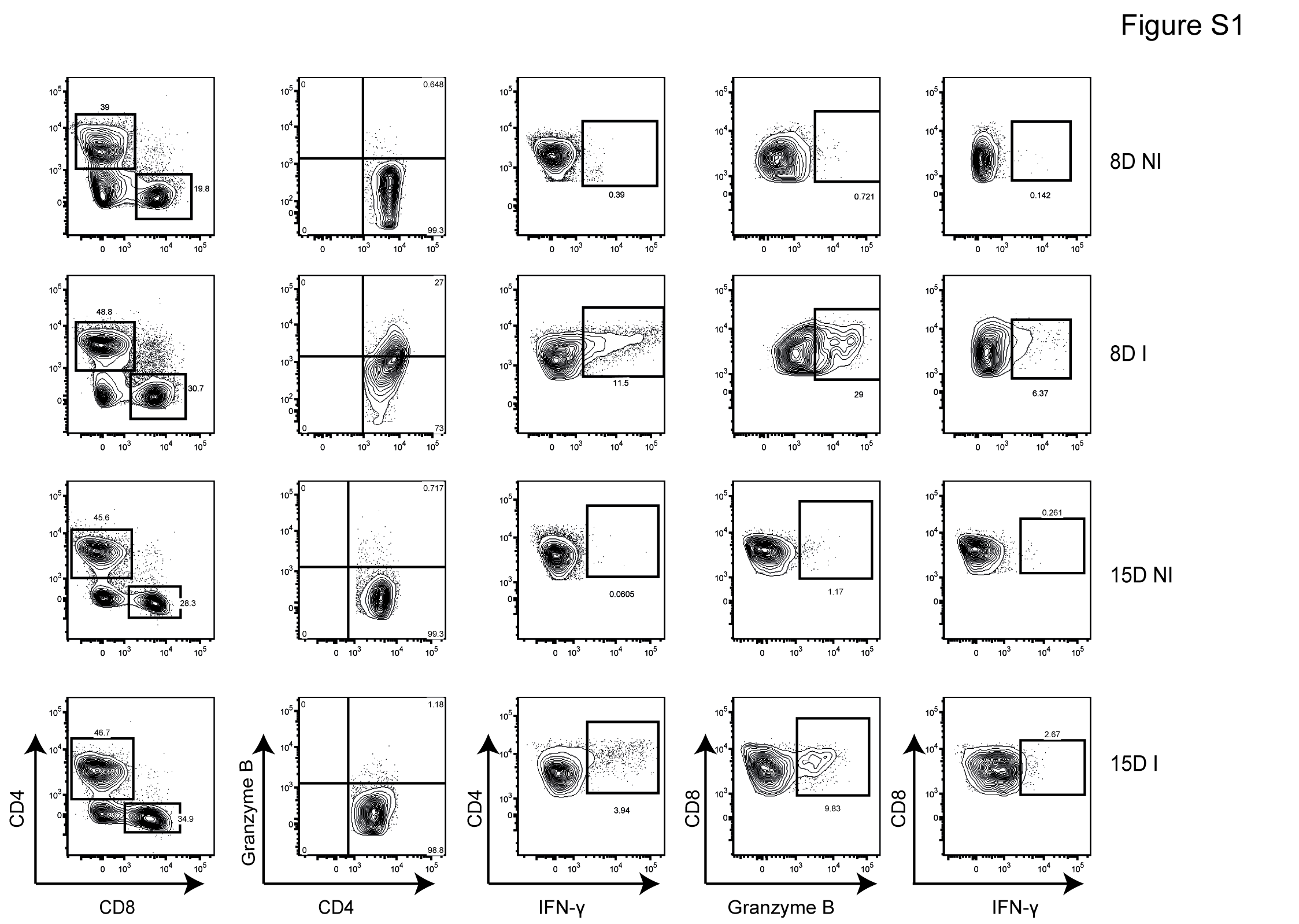

Supplement: Figure S1 — Identification of a GranzymeB+ CD4+T cell population in the axillary lymph nodes (ALNs) of Brucella-infected mice. CD4+ and CD8+ T cells from Brucella-infected C57BL/6 mice ALNs were analyzed by flow cytometry for the synthesis of IFN-γ and Granzyme B at 8 and 15 days post-infection. Numbers in outlined areas indicate percentages of cells. Data are representative of 3 separate experiments each involving groups of 5 mice. (TIF) [file pone.0082508.s001.tif]

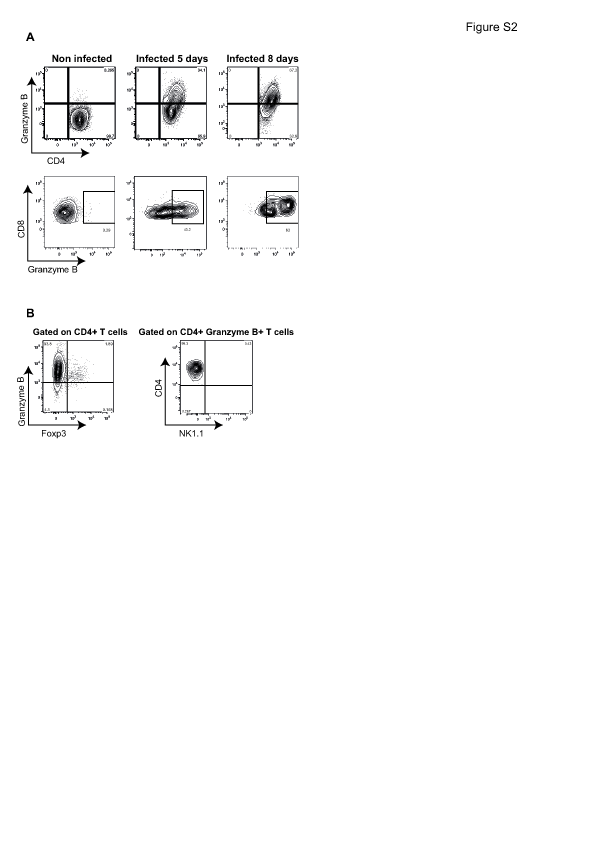

Supplement: Figure S2 — GranzymeB+ CD4+T cell population upon Brucella infection. (A) CD4+ and CD8+ T cells from the spleens of BALB/c mice infected by Brucella were analyzed by flow cytometry for the synthesis of IFN-γ and Granzyme B at 5 and 8 days post-infection. (B) Granzyme B-expressing CD4+ T cell population from Brucella-infected C57BL/6 mice was analyzed by flow cytometry for the expression of Foxp3 and NK1.1 at 8 days post-infection. Numbers in outlined areas indicate percentages of cells. Data are representative of 3 separate experiments each involving groups of 5 mice. (TIF) [file pone.0082508.s002.tif]

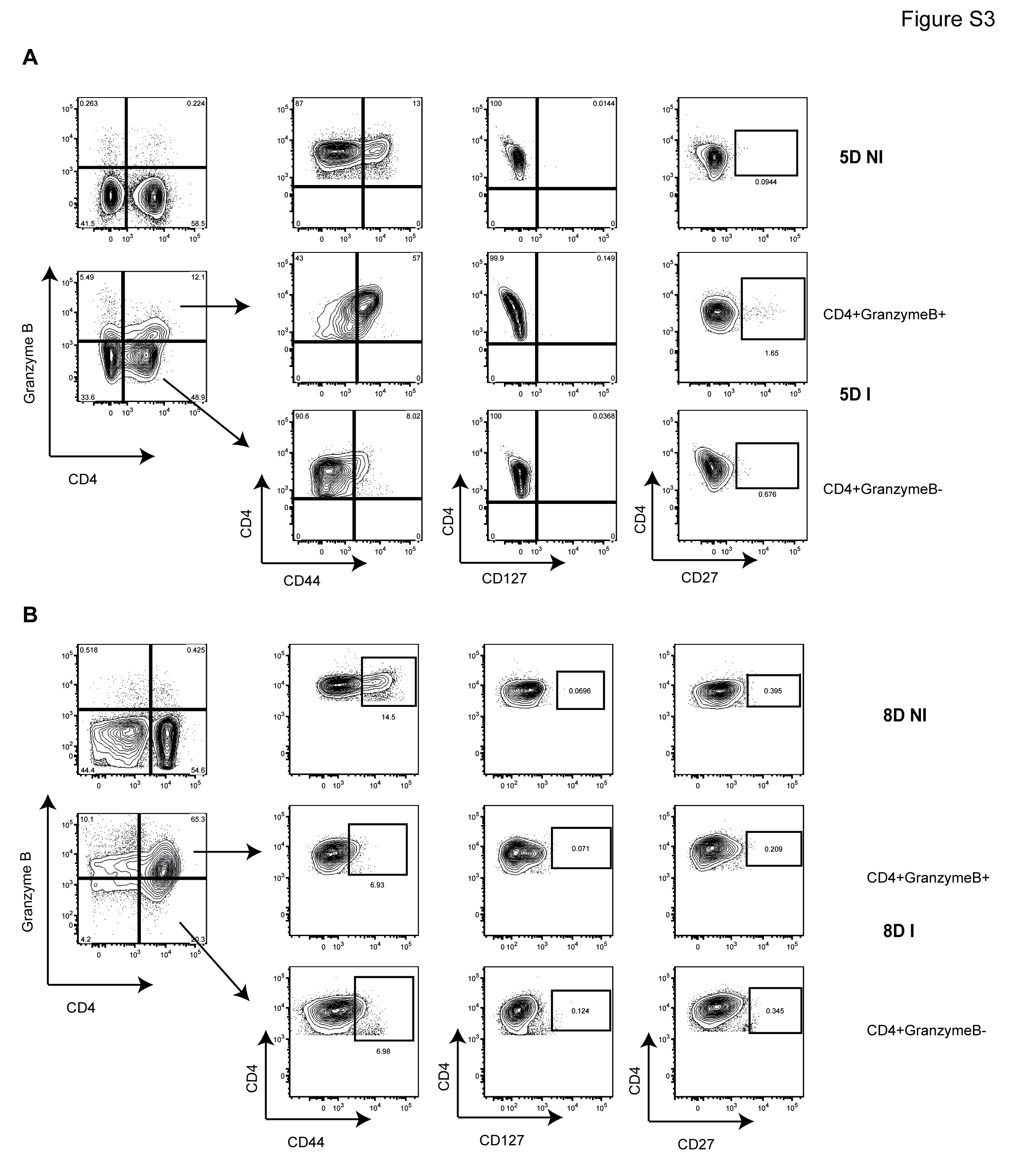

Supplement: Figure S3 — Characterization of CD4+ CTLs in Brucella infection model. The Granzyme B-expressing CD4+ T cell population from Brucella-infected C57BL/6 mouse spleens was analyzed by flow cytometry for the expression of CD44, CD127 and CD27 at 5 and 8 days post-infection. Numbers in outlined areas indicate percentages of cells. Data are representative of 3 separate experiments each involving groups of 5 mice. (TIF) [file pone.0082508.s003.tif]

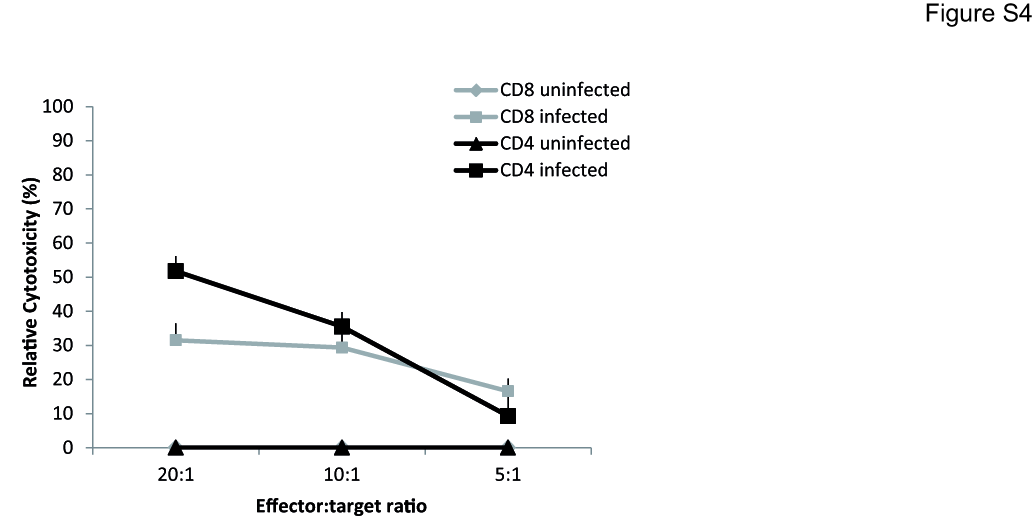

Supplement: Figure S4 — Functional characteristic of GranzymeB+ CD4+T cells. Macrophages were incubated with heat-killed B. abortus and cocultured with either CD8+ or CD4+ effector cells isolated from the spleens of BALB/c mice infected for 7 days with Brucella. Macrophage targets and effector cells were mixed at several effector:target /T ratios and incubated for 4 h. Effector cell-mediated cytotoxicity was analyzed by an LDH-release assay. Values given represent relative macrophage lysis as compared to a Tx-100 treated 100% control. (TIF) [file pone.0082508.s004.tif]

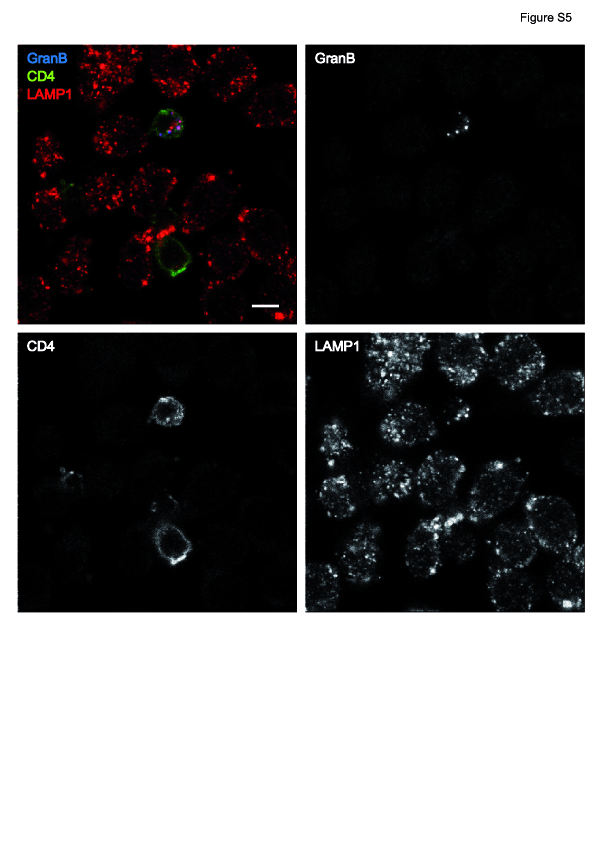

Supplement: Figure S5 — CD4 T-cell Granzyme B mostly localizes in LAMP1-positive vesicles. B. abortus-infected Raw macrophages were incubated for 1h with CD4 positive effector cells from the spleen of a mouse that had been infected with B. abortus for 8 days, fixed and processed for confocal fluorescence microscopy. Samples were stained for LAMP1, Granzyme B and CD4. White bar: 5 µm. (TIF) [file pone.0082508.s005.tif]
